# Supplementary material for: Transcriptional profiling and genetic analysis of a cystic fibrosis airway-relevant model shows asymmetric responses to growth in a polymicrobial community
Source: Microbiol Spectr. 2023 Sep 29;11(5):e02201-23. doi: 10.1128/spectrum.02201-23 (PMC10580927; doi:10.1128/spectrum.02201-23)
Supplement: Supplementary file 1 [file spectrum.02201-23-s0001.pdf]

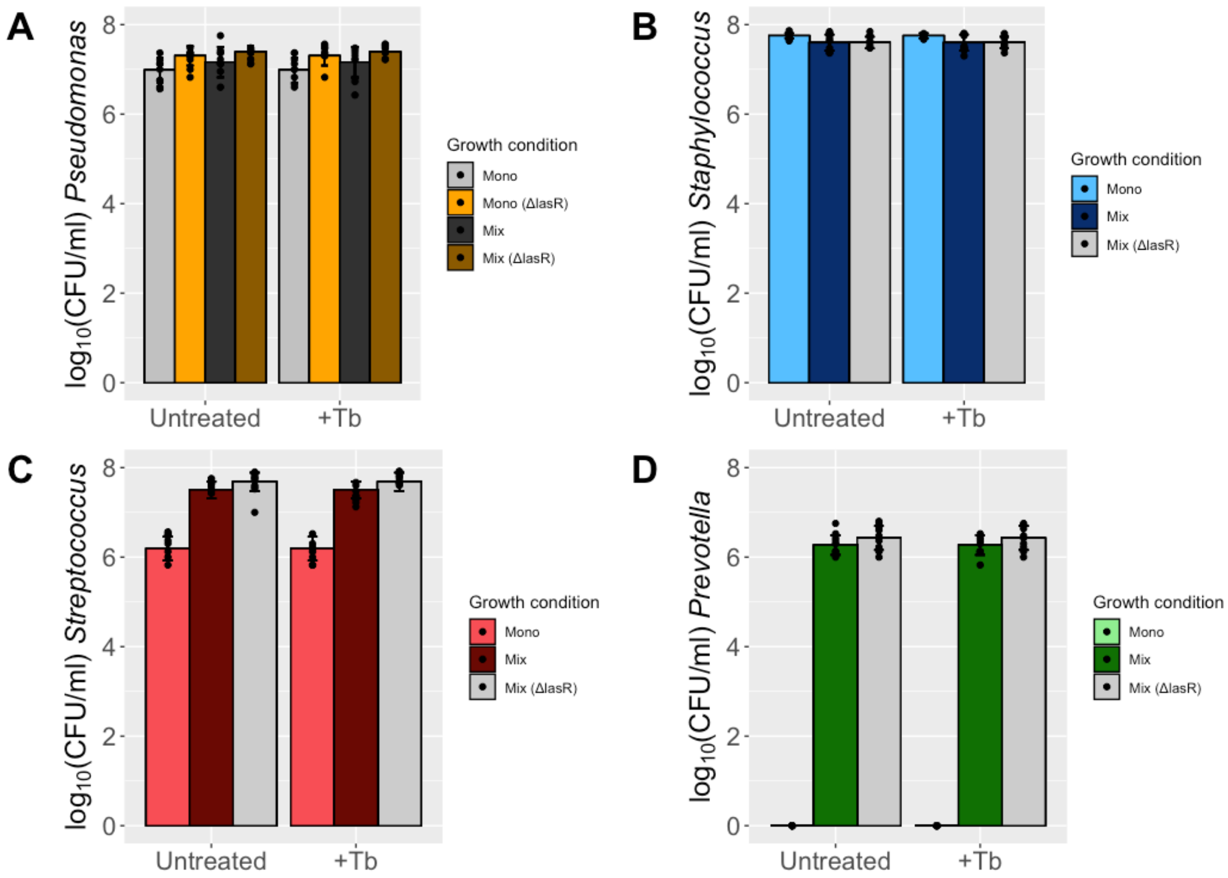

Figure S1. Acute exposure to 100ug/ml tobramycin does not impact bacterial viability. To confirm that viability was not impacted by an acute 15-minute exposure of tobramycin, CFU/ml counts of both monoculture and polymicrobial cultures exposed to fresh medium or fresh medium supplemented with 100µg/ml tobramycin were performed. **A**. Impact of tobramycin on *P. aeruginosa* mono and co-cultures. **B**. Impact of tobramycin on *S. aureus* mono and co-cultures. **C**. Impact of tobramycin on *S. sanguinis* mono and co-cultures. **D.** Impact of tobramycin on *P. melaninogenica* mono and co-cultures. CFU counts were not altered by the presence of 100 µg/ml tobramycin in any of the growth conditions. Three biological replicates with 3 technical replicates were performed, mean values were used to produce the bars, with standard deviation plotted as error. Individual datapoints are overlayed on the graphs. An ANOVA with a post hoc Bonferroni correction was performed on each of the datasets. For all datasets $p>0.05$.
